# High-throughput combinatorial screening of multi-component electrolyte additives to improve the performance of Li metal secondary batteries

**DOI:** 10.1038/s41598-019-42766-x

**Published:** 2019-04-17

**Authors:** Shoichi Matsuda, Kiho Nishioka, Shuji Nakanishi

**Affiliations:** 1 Global Research Center for Environment and Energy based on Nanomaterials Science, National Institute of Material Science, 1-1 Namiki, Tsukuba, Ibaraki 305-0044 Japan; 2 Graduate School of Engineering Science, Osaka University, 1-3 Machikaneyama, Toyonaka, Osaka 560-8531 Japan; 3 Research Center for Solar Energy Chemistry, Osaka University, 1-3 Machikaneyama, Toyonaka, Osaka 560-8531 Japan

**Keywords:** Batteries, Characterization and analytical techniques, Batteries

## Abstract

Data-driven material discovery has recently become popular in the field of next-generation secondary batteries. However, it is important to obtain large, high quality data sets to apply data-driven methods such as evolutionary algorithms or Bayesian optimization. Combinatorial high-throughput techniques are an effective approach to obtaining large data sets together with reliable quality. In the present study, we developed a combinatorial high-throughput system (HTS) with a throughput of 400 samples/day. The aim was to identify suitable combinations of additives to improve the performance of lithium metal electrodes for use in lithium batteries. Based on the high-throughput screening of 2002 samples, a specific combination of five additives was selected that drastically improved the coulombic efficiency (CE) of a lithium metal electrode. Importantly, the CE was remarkably decreased merely by removing one of these components, highlighting the synergistic basis of this mixture. The results of this study show that the HTS presented herein is a viable means of accelerating the discovery of ideal yet complex electrolytes with multiple components that are very difficult to identify via conventional bottom-up approach.

## Introduction

There are currently increasing demands for safe electrical energy storage devices having high energy density and long lifespans for use in electric vehicles and smart grid systems, so as to make efficient use of renewable energy sources. Lithium ion secondary batteries are one such energy storage device. These units have many components, including active electrode materials, polymeric porous separators and electrolyte solutions. In addition, some of the constituent parts of batteries are themselves multi-component systems. It is well known that the performance of the cathode material in lithium ion batteries greatly depends on the ratio of the constituent elements as well as the type and concentration of dopants^1,2^. Furthermore, recent work has shown that certain combinations of chemicals can form a solid electrolyte interface (SEI) that functions as the negative electrode^3–7^. Thus, optimal combinations of these electrode components need to be identified out of many potential candidates, with the aim of developing batteries for practical applications.

Data-driven experimental design is one of most promising approaches to identifying suitable electrode compounds, based on techniques such as evolutionary algorithms or Bayesian optimization^7–9^. In addition, because the reliability and accuracy of predictions generated by algorism are highly correlated with the size and quality of the experimental data set, a high-throughput screening system (HTS) must be developed. To date, various HTS approaches have been applied to identifying or characterizing novel battery components. Watanabe *et al*. identified a novel positive electrode material having the required functionality using an HTS method based on automated robotics^10–12^. Dahn *et al*. also ascertained the optimal metal alloys for use as negative electrode materials using combinatorial synthesis based on a sputtering technique coupled with a multi-channel battery evaluation system^13–16^. Although these studies successfully demonstrated the effectiveness of HTS approaches with regard to selecting solid-state battery materials, this technique would seem to have limited usefulness in the screening of liquid electrolytes, unless these electrolytes are also complex multi-component mixtures, such as combinations of lithium salts, solvents and additives.

As noted above, various combinations of additives can produce suitable SEI^3–7^. The identification of the most appropriate combination could be performed using an HTS-based approach. Unfortunately, such methods have not yet been applied for this purpose, due to difficulties in simultaneously performing automated sequential electrochemical operations based on robotics engineering together with big-data processing and highly sensitive analyses. Automated sequential electrochemical operation is essential so as to obtain data sets that are sufficiently large (comprising at least several hundred data points) to allow the use of various data-driven methods. In the present work, we developed an HTS method with the aim of identifying optimal combinations of additives to improve the performance of lithium metal electrodes.

## Results

### Development of high-throughput battery evaluation system

In the present work, we developed multi-channel electrochemical cells for the HTS approach based on a microplate technique that has often been used in the field of biochemical research. Although the utilization of microplates for electrochemical analysis has been reported previously^17^, there are no examples in the literature of the application of such plates to the study of lithium ion batteries. The microplate-based electrochemical cell (hereafter termed the *E-microplate*) was fabricated by placing the battery components, including a positive electrode, negative electrode, separator and electrolyte solution, inside sample wells (Fig. S1A). The plate itself was constructed of polypropylene and had 96 wells in which electrochemical assemblies could be constructed. The applicability of the *E-microplate* to the evaluation of battery performance was initially assessed by conducting the electrochemical deposition/stripping of metallic lithium on nickel foil, and typical results originated from lithium deposition/stripping in this electrochemical setup are presented in Fig. S1B. These data demonstrate that the *E-microplate* could indeed be utilized to evaluate battery performance.

**Figure 1:**
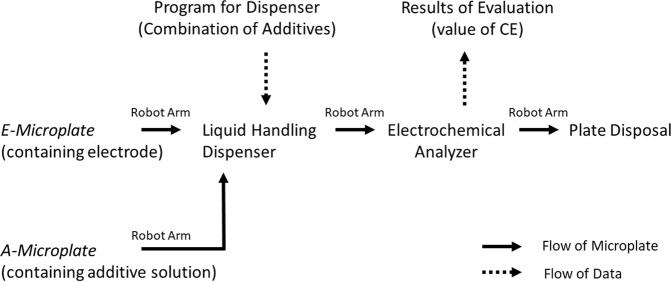
Schematic illustration of the experimental protocol associated with the HTB system.

The high-throughput battery evaluation system consisted of a liquid handling dispenser and a 96 channel electrochemical analyzer equipped with a robotic arm for transporting microplates (hereafter termed the HTB-system). This apparatus was constructed in an Ar-filled glovebox (Fig. S2). The operational protocol of the HTB-system is summarized schematically in Fig. 1. In this process, two types of microplates (*E-microplates* and *A-microplates* containing additive solutions) were placed on stacking racks. Each *E-microplate* was transported from the stacking rack to the liquid handling position by the robotic arm and the electrolyte containing the additives was injected from the *A-microplate* into each well of the *E-microplate* by the liquid dispenser. The *E-microplate* was subsequently transported to the electrochemical analyzing position and the battery performance was evaluated. After these measurements, the *E-microplate* was disposed of and a new series of experiments was initiated in the same manner. By repeating this procedure, a throughput of 400 samples/day was achieved.

### Combinatorial CE evaluation in the _14_C_5_ system

**Figure 2:**
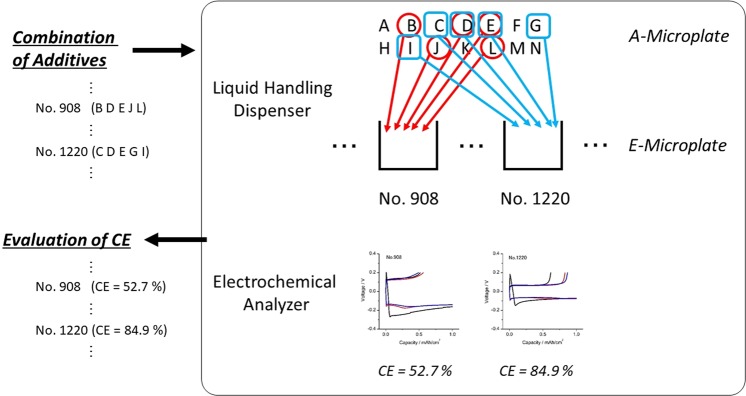
Schematic illustration of the experimental protocol associated with combinatorial CE evaluation in the _14_C_5_ system.

Next, we attempted to identify a combination of additives that would improve the performance of the lithium deposition/stripping reaction, using the newly developed HTB-system. In these experiments, five different additives were selected from the list of fourteen chemical compounds shown in Table 1, all of which are representative lithium salts and other additives previously described in the literature. Employing a method already described in a prior paper^18^, the coulombic efficiency (CE) of each cell containing five additives was quantitatively evaluated according to the protocol shown in Fig. 2. As an example, in the case of sample 1220 in Table 2, an electrolyte solution containing LiAsF_6_ (labeled C in Table 1), LiClO_4_ (D), LiBOB (E), LiBr (G) and LiF (I) was prepared by the liquid handling dispenser. The average CE value in the second and third cycles for this sample was determined to be 84.9%. Here, we should mentioned about the accuracy of electrochemical data obtained by HTB-system. The twelve different experiments were carried out for CE evaluation (i.e. twelve experiments performed on twelve different *E-microplate* with different well position) and average standard deviation is less than 5% (Fig. S3). These results demonstrate the high accuracy for CE obtained by HTB-system.

**Table 1:** The potential additives considered in this work.

	Additive	Concentration
A	LiPF_6_	2 wt%
B	LiBF_4_	2 wt%
C	LiAsF_6_	2 wt%
D	LiClO_4_	2 wt%
E	LiBOB	1 wt%
F	Li_3_PO_4_	1 wt%
G	LiBr	2 wt%
H	LiCl	1 wt%
I	LiF	1 wt%
J	PC	10 v%
K	DEC	10 v%
L	DMC	10 v%
M	VC	10 v%
N	FEC	10 v%

**Table 2:** Ten best-performing combinations.

Additives					Sample No.	Coulombic efficiency			
						1^st^	2^nd^	3^rd^	Ave. (2^nd^ & 3^rd^)
**Example**									
—	—	—	—	—	control	43.4	74.4	74.7	74.5
B	D	E	L	J	908	51.7	56.3	49.1	52.7
C	D	E	G	I	1220	61.8	83.1	86.7	84.9
**Top 10 sample**									
D	E	G	L	N	1588	64.2	87.4	89.7	88.6
F	G	K	L	N	1839	56.5	86.3	90.2	88.2
D	E	J	M	N	1620	70.6	88.3	87.8	88.1
B	D	E	F	J	884	65.1	86.3	89.2	87.8
B	E	I	J	L	1066	74.6	87.1	87.7	87.4
A	E	H	L	N	568	50.6	85.8	89.1	87.4
B	J	L	M	N	1209	72.4	86.1	88.5	87.3
A	D	F	K	N	446	65.5	85.9	88.5	87.2
D	J	K	L	N	1747	60.9	85.6	88.8	87.2
A	C	D	F	L	236	46.3	86.8	87.4	87.1

The average CE values obtained from the second and third cycles are shown in the rightmost column.

**Figure 3:**
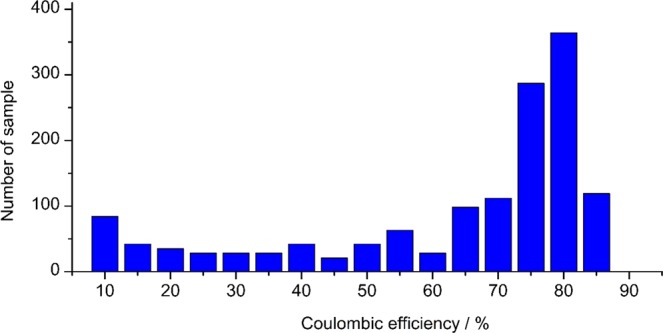
Histogram of the CE values of the 2002 (=_14_C_5_) samples.

**Figure 4:**
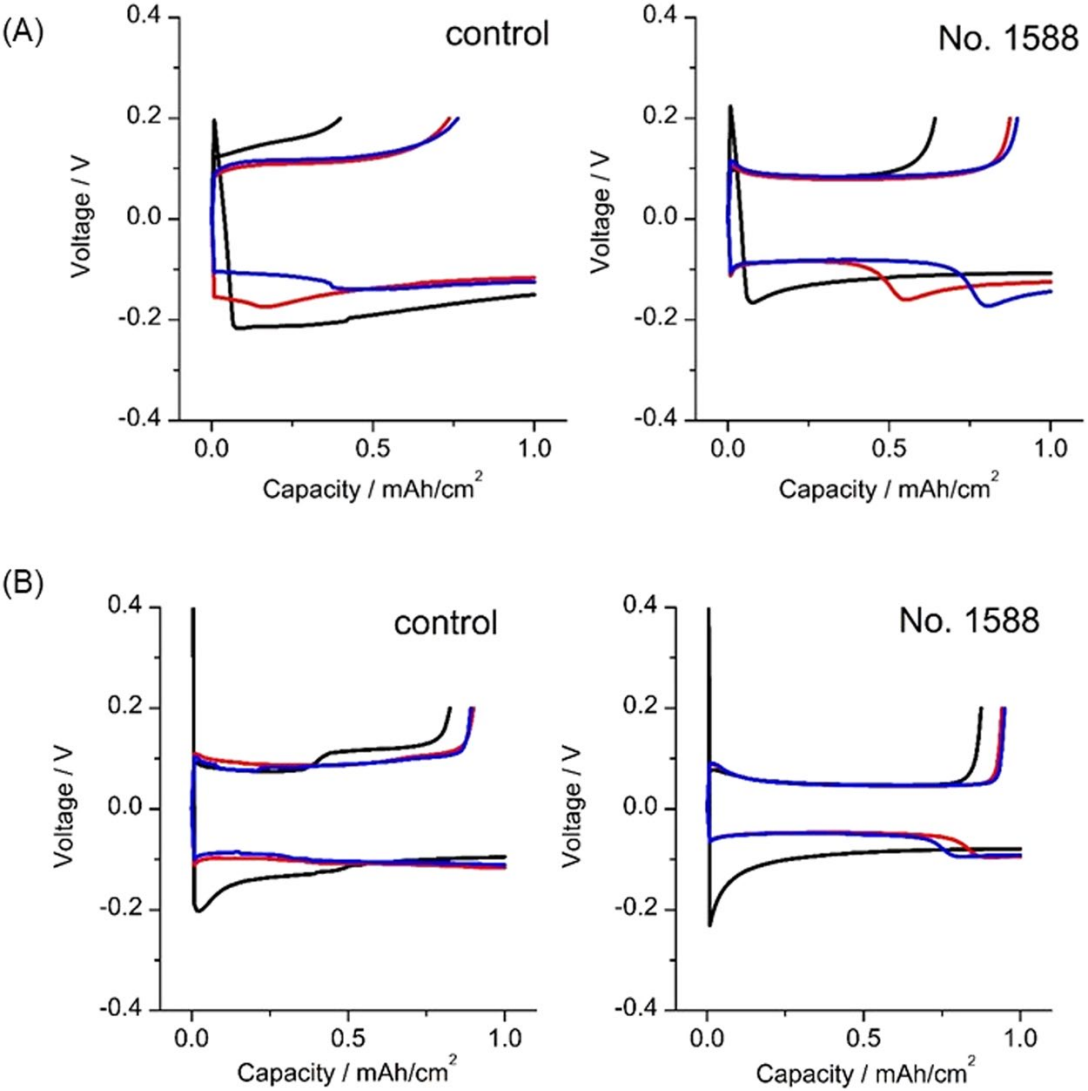
Electrochemical profiles of Li deposition/stripping cycles. (**A**) Using the HTB-system and (**B**) using 2032 coin-type cells. Black, red and blue curves indicate data from the first, second and third cycles, respectively.

All the possible combinations of five additives from the fourteen chemical compounds (_14_C_5_ = 2002) were assessed using the automated HTB-system and the results are summarized in the histogram in Fig. 3, while the top twenty samples are presented in Table 2. The data show that the specific combination of additives labeled 1588 (LiClO_4_, LiBOB, LiBr, DMC and FEC) exhibited the highest CE, with values up to 88.6% (Fig. 4A). Importantly, this combination of additives discovered by HTB-system also showed a superior CE even in a 2032 coin-type cell (Fig. 4B). These results clearly demonstrate that the HTB-system was able to rapidly identify the optimal combination of additives for use in lithium batteries. It should be mentioned that the CE evaluation experiment in Fig. 1 was conducted at relatively high current density condition (3 mA/cm^2^). Thus, some of the cell was short circuited by the formation of dendritic lithium during repeated lithium deposition/stripping process with certain probability. Such cell showed the relatively low CE value less than 20%. Such undesired formation of dendritic lithium can be suppressed by optimized design of *E-microplate*, such as electrode configuration, amount of electrolyte and applied confining pressure. The trial relating with these issues is now on-going in our laboratory.

To determine whether or not all of these five additives were required to obtain a high CE, we prepared 32 different electrolytes, each of which lacked one or more of the five additives. The various combinations of the additives that were assessed and the corresponding CE values are summarized in Table S1. Of particular note is that the CE was significantly reduced when any one of the five additives was missing (samples (ii) to (vi) in Table S1). For several additive combinations, we also carried out the CE evaluation by coin-type cell (samples (vii) and (viii) in Table S1). As a result, a trend similar to that of HTB-system was observed (Fig. S4). Thus, the enhanced CE was only obtained when all five additives were employed (sample (i)), suggesting that these compounds worked in a cooperative fashion.

### Analyses of SEIs

**Figure 5:**
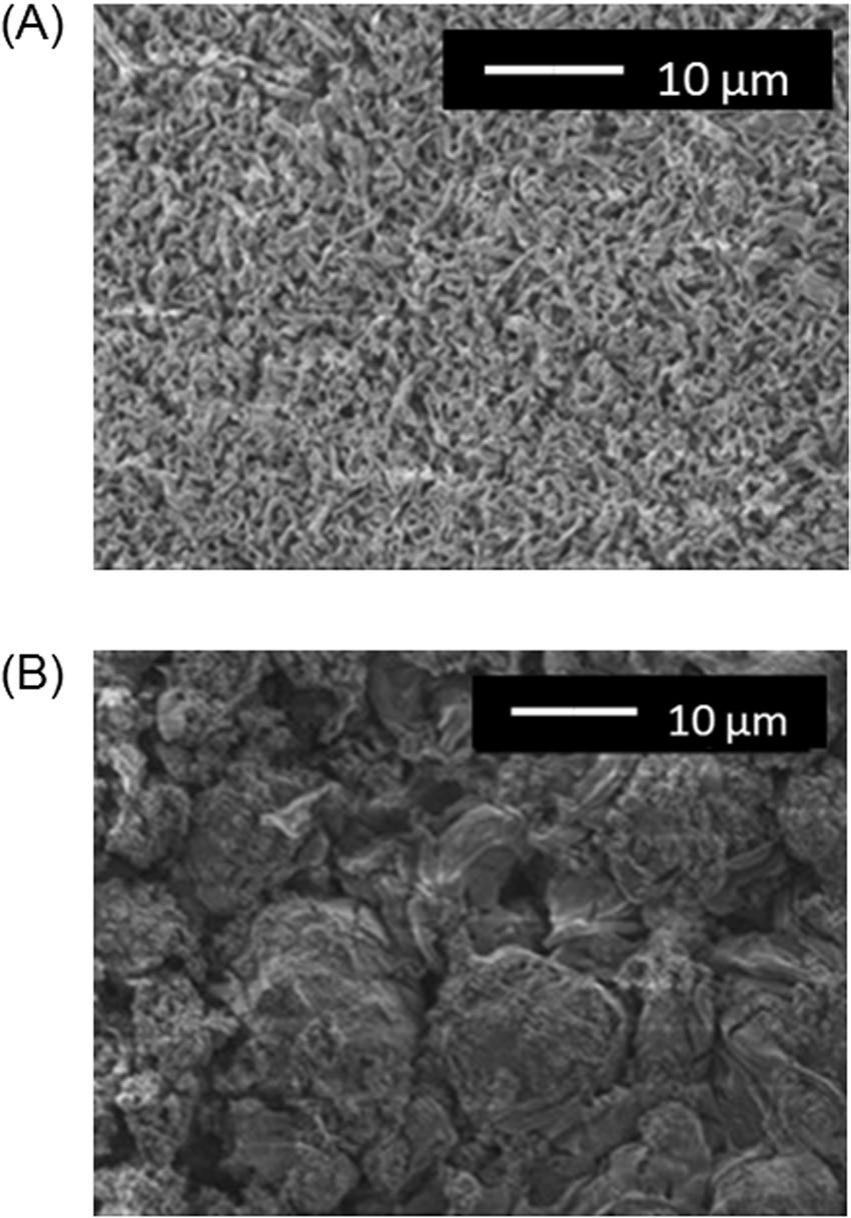
Top-view SEM images of the lithium electrodes. (**A**) Sample (i) and (**B**) sample (x) in Table S2. The lithium electrodes were removed from the electrochemical cell after lithium metal deposition at a capacity of 3.0 mAh/cm^2^.

The physicochemical factors determining the improved CE were examined by characterizing the SEIs formed in the presence of all five additives (sample (i), Table S1) and in electrolytes lacking one of the five additives (samples (ii) to (vi)) or in an additive-free electrolyte (sample (x)). These characterizations were performed by scanning electron microscopy (SEM), X-ray photoelectron spectroscopy (XPS) and attenuated total reflectance Fourier transform infrared spectroscopy (ATR-FTIR). Figures 5 and S5 present SEM images of the surfaces of the lithium electrodes after lithium metal deposition at a capacity of 3 mAh/cm^2^. A comparison of samples (i) and (x) shows that the presence of all five additives resulted in smaller, more uniform deposits, thus improving the macroscopic flatness of the SEI surface (Fig. S6). In contrast, samples lacking one of the five additives (samples (ii) to (vi)) showed reduced uniformity of the deposit size, as can be seen in Fig. S5. Notably, sample (iv) (which did not contain LiBr) exhibited the most inhomogeneous and rough surface (Fig. S5C), and its uniformity was inferior to that of the sample formed in an electrolyte containing only LiBr as the additive (sample (vii), Fig. S5F). A comparison between samples (i) and (ix) demonstrates that the homogeneity could be further improved by incorporating other components in addition to LiBr.

**Figure 6:**
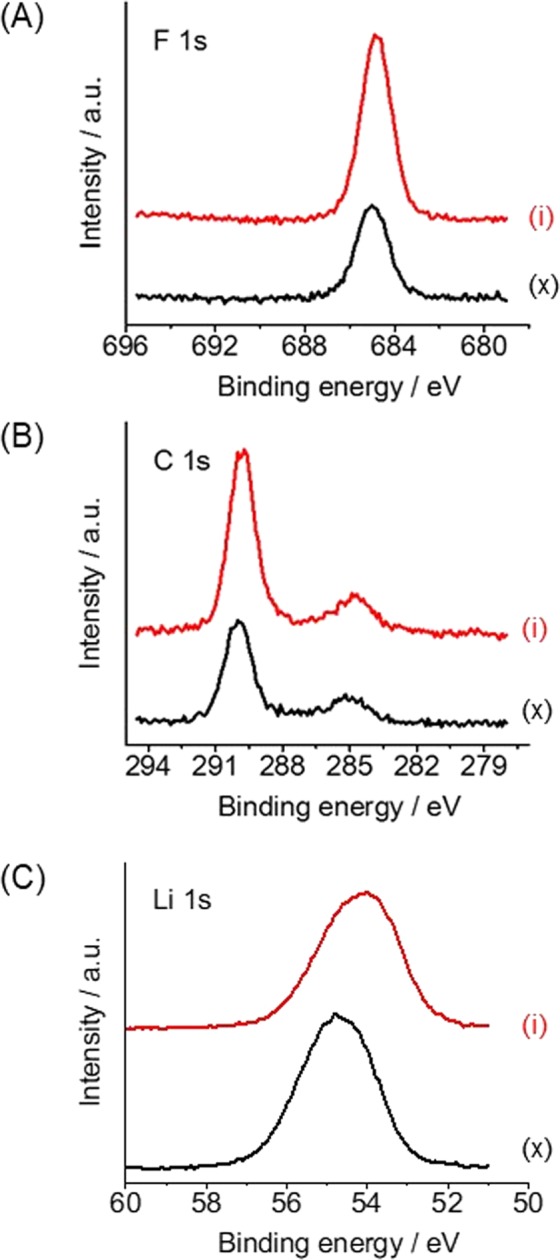
XPS spectra of samples (i) and (x). The samples were removed from the electrochemical cell after lithium metal deposition at a capacity of 3.0 mAh/cm^2^. (**A**) F 1 s, (**B**) C 1 s and (**C**) Li 1 s regions. Red and black curves indicate data for samples (i) and (x), respectively.

The chemical compositions of the SEIs were examined by XPS. Sample (i) generated a clear peak at 685.0 eV in the F 1 s region (Fig. 6A) that was assigned to LiF^19^. Although this same peak was observed in the patterns of the other samples (Fig. S7), the intensity of the peak produced by sample (i) was higher than that from sample (x). In contrast, samples (i) to (vi) produced peaks at 290.1 and 295.0 eV in the C 1 s region, with sample (i) giving the most intense peaks (Fig. 6B). ATR-FTIR analyses showed that the peaks assignable to C=C bonds and to Li_2_CO_3_ were significantly higher for sample (i) as compared to sample (x) (Fig. S8). Based on literature reports^20^, it appears that the organic compounds in these specimens were formed via the reductive decomposition of the electrolyte and/or additives based on the strong reducing power of metallic lithium. In addition to these intense peaks, other peaks likely due to decomposition products of the additives and/or lithium salts were seen in the B 1 s, N 1 s, S 2p, Cl 2p and Br 3d regions (Fig. S9). However, these peaks were small and it is likely that these components did not significantly contribute to the quality of the SEI and hence to the improvement of the CE value. Finally, we examined the depth profiles in the C 1 s and Li 1 s regions of the SEIs generated by samples (i) and (x). The XPS spectra obtained after Ar ion sputtering for 31 min corresponded to a depth more than 238 nm (7.7 nm min^−1^ × 31 min) below the surface^21^. The resulting depth profiles did not show any notable changes regardless of the presence of additives (Fig. S7).

## Discussion

Let us discuss about the origin of improved CE in the case of sample (i), which contained all five additives (LiClO_4_, LiBOB, LiBr, DMC and FEC). The result of SEM observation revealed that the improved homogeneity of SEI. Actually, FEC and LiBOB were reported to contribute to the formation of compact and uniform SEI with improved morphology^22–24^. In addition, the improved morphological homogeneity of the SEI surface in the presence of LiBr has also recently reported^25^. The synergetic effect of these compounds results in the formation of compact and uniform SEI.

The chemical compositions of the SEI is also essential factor for determining CE. As revealed by XPS analysis, SEI generated by sample (i) contains much amount of LiF and organic compounds assignable to Li_2_CO_3_ and/or oligomers (as the decomposition products of the electrolyte). The formation of LiF rich SEI in the presence of FEC is well known in the literature^22–24^. On the other hands, the formation process of the organic compounds in the SEI is much complicated because the electrolyte contains large number of carbon containing species. Among them, DMC is known to form organic compound rich SEI by its reductive decomposition^26^. These compound contribute to generate high quality SEIs that in turn improve the CE value. However, general guidelines for selecting optimum electrolyte components that ensure a high-quality SEI have not been established. The fact that a good SEI was formed from this complex electrolyte system containing five additives is important. These results indicate that a wide range of suitable electrolyte systems composed of multiple different salts, solvents and additives are still awaiting discovery.

## Conclusions

In the present study, we developed a combinatorial high-throughput system with a screening rate of 400 samples/day, specialized for the evaluation of lithium battery components. Using this system, a specific combination of five chemical compounds was identified that improved the CE value during lithium deposition/stripping cycles. Analyses of the SEI film formed in this new electrolyte showed large amounts of lithium fluoride and lithium organic compounds that are known to enhance CE. Importantly, the CE was greatly decreased simply by the lack of one of the five additives, suggesting the cooperative effect of the five compounds. The results obtained in the present study confirm that the utilization of this HTB-system can accelerate the discovery of improved electrolyte compositions. While it is time-consuming or even unrealistic to find ideal combinations of multiple additives using a traditional bottom-up approach, the technique presented herein efficiently identifies complex electrolytes that can lead to superior lithium battery performance.

## Methods

### Chemicals

Lithium bis(trifluorosulfonyl)imide (LiTFSI), LiPF_6_, LiBF_4_, 1,2-dimethoxyethane (DME), 1,3-dioxolane (DOL), propylene carbonate (PC), diethyl carbonate (DEC), dimethyl carbonate (DMC), vinylene carbonate (VC) and fluoroethylene carbonate (FEC) were purchased from KISIDA Chemical. LiAsF_6_, LiClO_4_, Li_3_PO_4_, LiNO_3_, LiBr, LiCl, LiF and lithium bis(oxalate)borate (LiBOB) were purchased from Sigma Aldrich and used after drying at 100 °C for 15 h under vacuum.

### Electrochemical characterization of lithium metal deposition/stripping

The cells employed during electrochemical characterization consisted of nickel foil ($\varnothing$: 6 mm for *E-microplate* type cell, $\varnothing$: 16 mm for 2032 coin type, 30 μm thickness, Nilaco Corp.) as the working electrode and metallic lithium foil ($\varnothing$: 6 mm for *E-microplate* type, $\varnothing$: 16 mm for 2032 coin type cell, 400 μm thickness; Honjo Metal Co., Ltd.) as the counter electrode. Glass filters ($\varnothing$: 8 mm for *E-microplate* type, $\varnothing$: 20 mm for 2032 coin type, GF/A, Whatman) were used as separators after drying at 60 °C for 15 h under vacuum. A solution of 1 M LiTFSI in a DME:DOL mixture (1:1 by volume) also containing 1 wt% LiNO_3_ was employed as the electrolyte. All solvents were dehydrated with molecular sieves (3 A, 1/8 in., Wako Pure Chemical Industries, Ltd.) before use. Elecrolyte amount of 50 μL or 100 μL was injected into for *E-microplate* or 2032 coin type cell, respectively. The applied confining pressure in each well of *E-microplate* was at around 0.1 MPa. Galvanostatic experiments were conducted with a battery charging/discharging device (HJ1020mSD8, Hokuto Denko Corp.).

### SEM, XPS and ATR-FTIR analyses

Scanning electron microscopy (SEM, JSM-7800F, Jeol), X-ray photoelectron spectroscopy (XPS, Axis Ultra, Kratos Analytical Co.) with monochromated Al Kα X-rays (hν = 1486.6 eV) and Fourier transform infrared spectroscopy (FTIR, Nicolet iS50, Thermo Scientific) were used to characterize the electrodes. Argon ion etching (acceleration voltage = 2 keV, ion beam current = 20 mA) was applied to obtain depth profiles. Prior to these analyses, the electrodes were removed from the electrochemical cells, washed three times with DME and dried under vacuum. The samples were not exposed to the ambient atmosphere during the preparation procedures.

## Supplementary information

Supporting Information
