# Supplementary material for: High-throughput combinatorial screening of multi-component electrolyte additives to improve the performance of Li metal secondary batteries
Source: Sci Rep. 2019 Apr 17;9:6211. doi: 10.1038/s41598-019-42766-x (PMC6470175; doi:10.1038/s41598-019-42766-x)
Supplement: Supplementary file 1 — Supporting Information [file 41598_2019_42766_MOESM1_ESM.docx]

**Supplementary Information**

**High-throughput combinatorial screening of multi-component electrolyte additives to improve the performance of Li metal secondary batteries**

*Shoichi Matsuda^1,*^, Kiho Nishioka^2^, and Shuji Nakanishi^2,3^*

^1^ Global Research Center for Environment and Energy based on Nanomaterials Science, National Institute of Material Science, 1-1 Namiki, Tsukuba, Ibaraki 305-0044, Japan

^2^ Graduate School of Engineering Science, Osaka University, 1-3 Machikaneyama, Toyonaka, Osaka 560-8531, Japan

^3^ Research Center for Solar Energy Chemistry, Osaka University, 1-3 Machikaneyama, Toyonaka, Osaka 560-8531, Japan

**
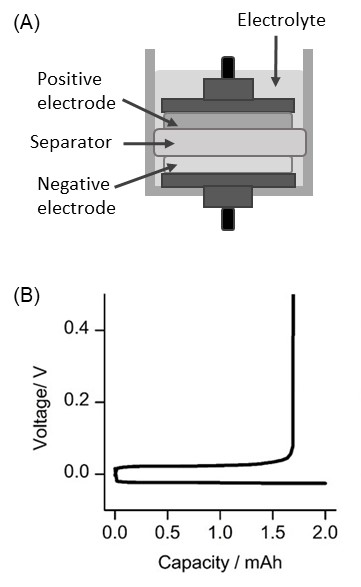
**

**Figure S1 Electrochemistry on *E-microplates***. (A) Schematic of a cell in the *E-microplate*. (B) A representative electrochemical profile obtained during Li deposition/stripping in the *E-microplate.*


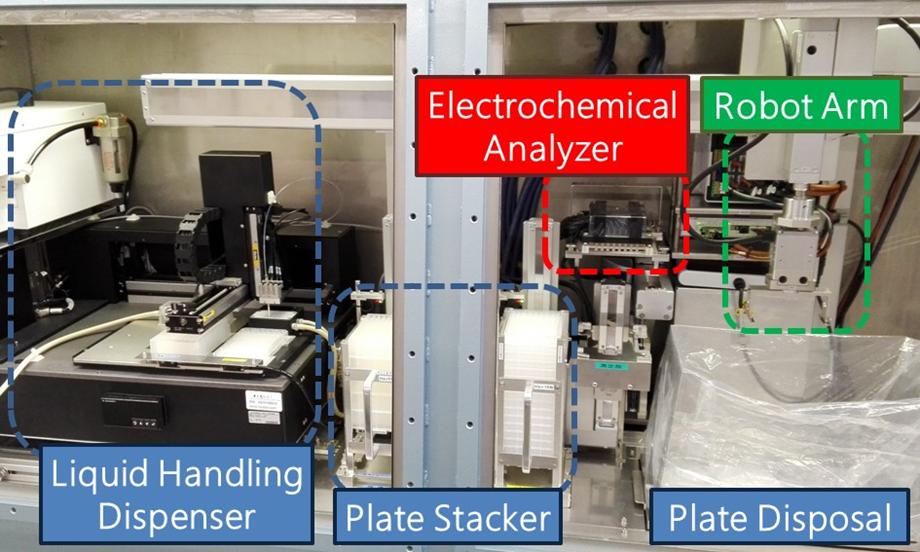


**Figure S2 Photographic image of the HTB-system developed in the present study.**


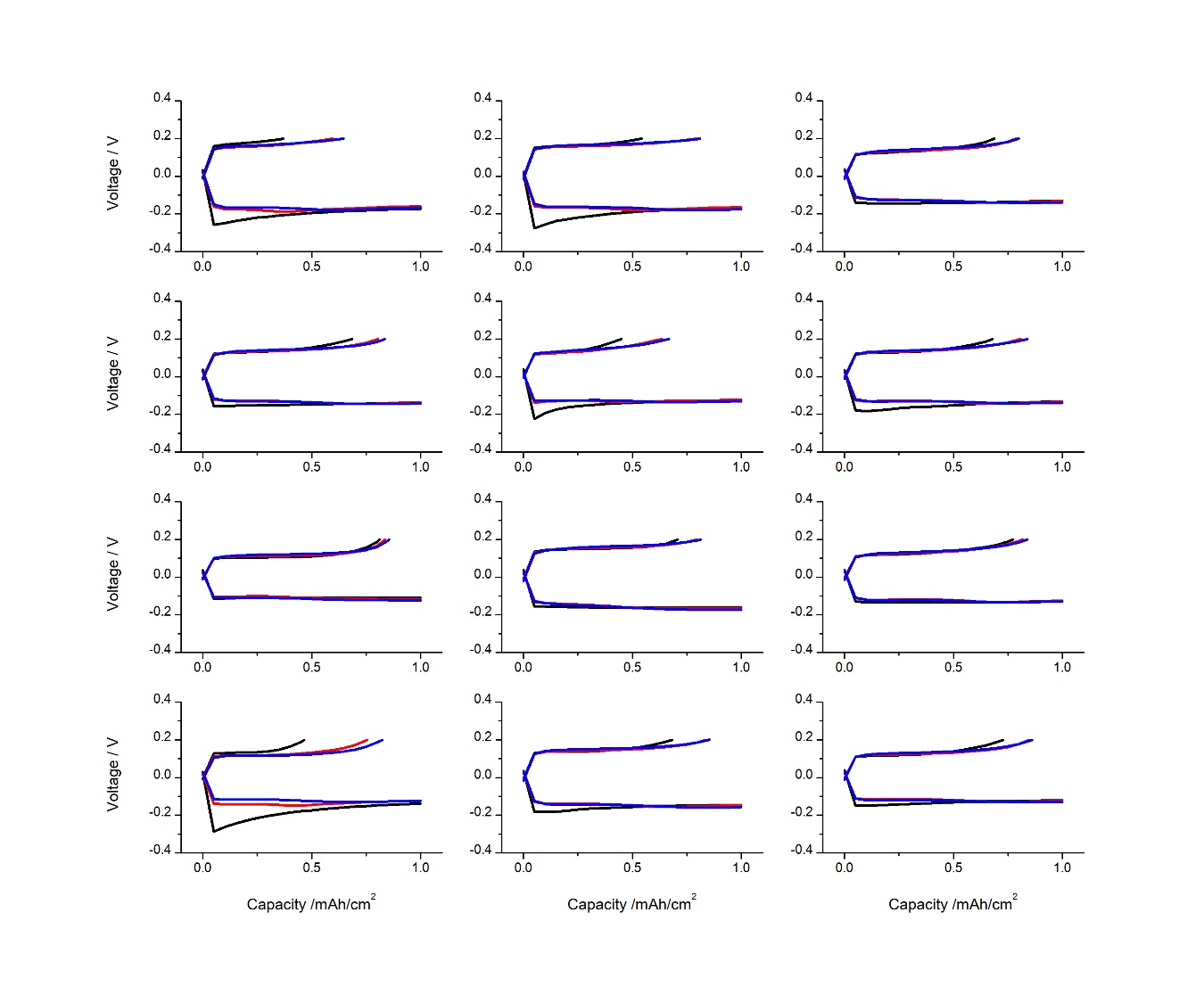


**Figure S3 Electrochemical profiles of Li deposition/stripping cycles.** Using the HTB-system. Black, red and blue curves indicate data from the first, second and third cycles, respectively. The twelve different experiments were carried out for CE evaluation (i.e. twelve experiments performed on twelve different *E-microplate* with different well position) and average standard deviation of CE is less than 5%.

**
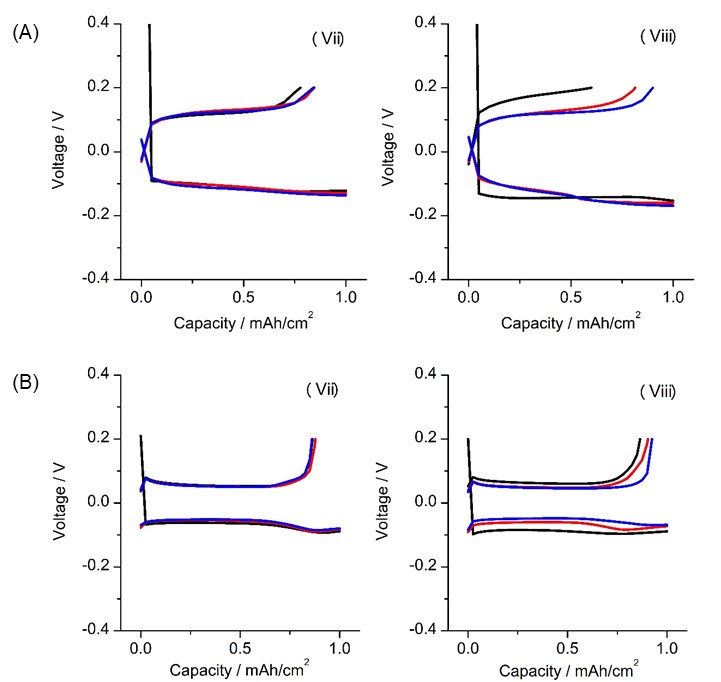
**

**Figure S4 Electrochemical profiles of Li deposition/stripping cycles.** (A) Using the HTB-system and (B) using 2032 coin-type cells. Black, red and blue curves indicate data from the first, second and third cycles, respectively.


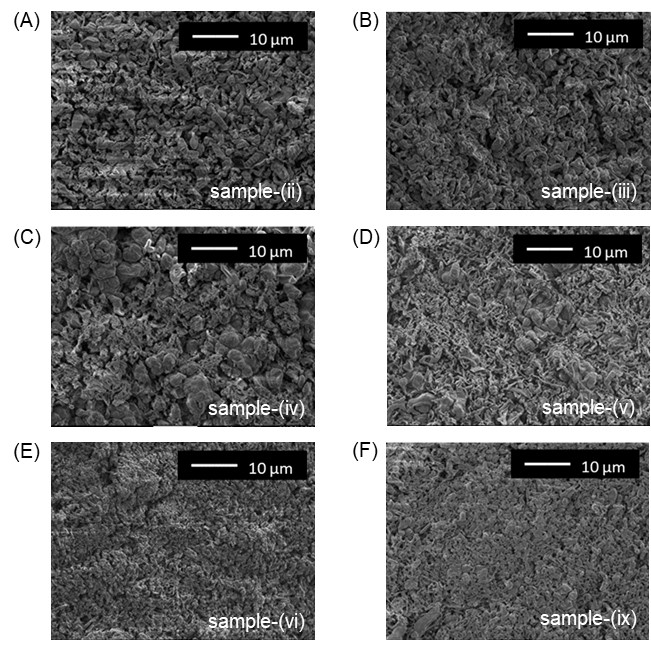


**Figure S5 Top-view SEM images of the lithium electrodes.** Samples (A) (ii), (B) (iii), (C) (iv), (D) (v), (E) (vi) and (F) (ix). The lithium electrodes were removed from the electrochemical cell after lithium metal deposition at a capacity of 3.0 mAh/cm^2^.


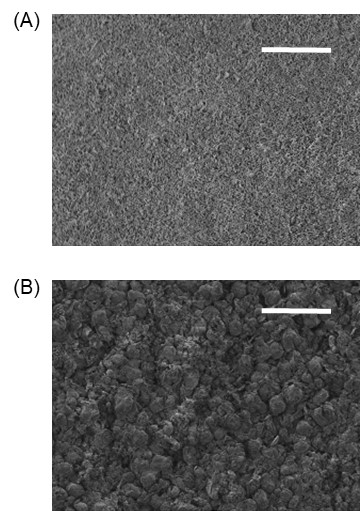


**Figure S6 Top-view SEM images of the lithium electrodes.** (A) Sample (i) and (B) sample (x) in Table S2. The lithium electrodes were removed from the electrochemical cell after lithium metal deposition at a capacity of 3.0 mAh/cm^2^. Scale bar is 50 μm.


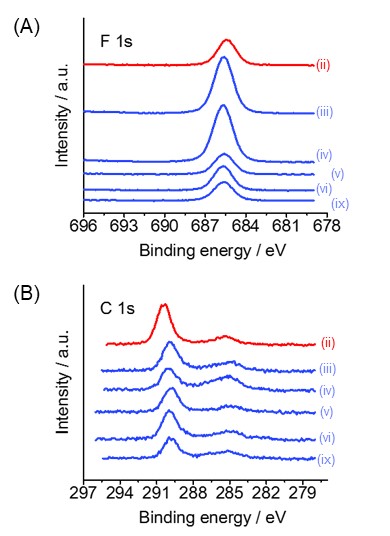


**Figure S7 XPS spectra of samples (ii) to (vi) and (ix).** The samples were removed from the electrochemical cell after lithium metal deposition at a capacity of 3.0 mAh/cm^2^. (A) F 1s and (B) C 1s regions. The red and blue curves indicate data for samples (ii) and samples (iii) to (vii).


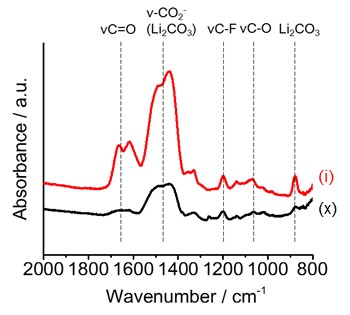


**Figure S8 ATR-FTIR spectra of samples (i) and (x).** The samples were removed from the electrochemical cell after lithium metal deposition at a capacity of 3.0 mAh/cm^2^. Red and black curves indicate data for samples (i) and (x), respectively.


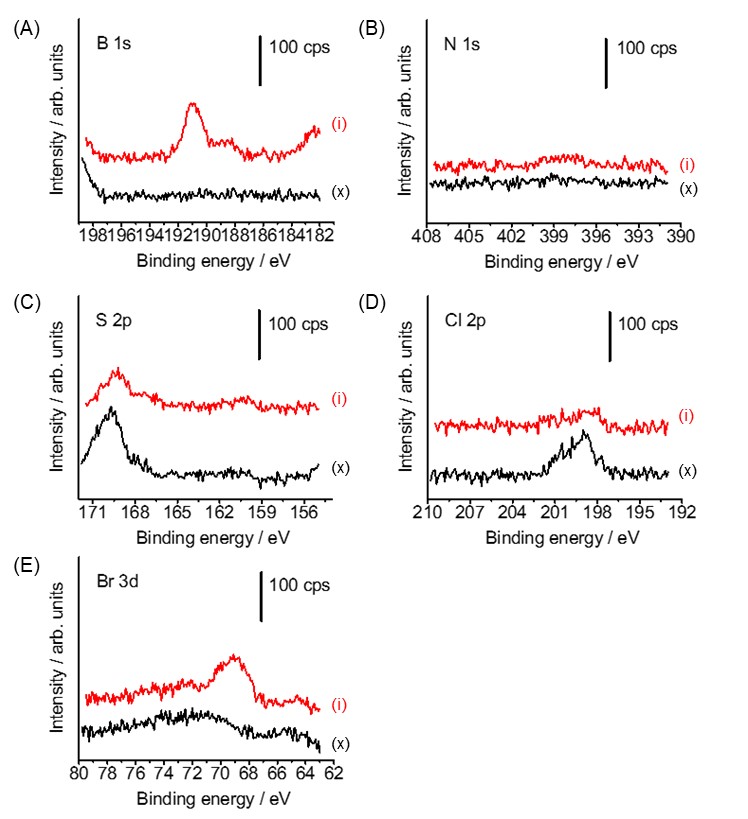


**Figure S9 XPS spectra of samples (i) and (x).** The samples were removed from the electrochemical cell after lithium metal deposition at a capacity of 3.0 mAh/cm^2^. (A) B 1s, (B) N 1s, (C) S 2p, (D) Cl 2p and (E) Br 3d regions. Red and black curves indicate data for samples (i) and (x), respectively.

**
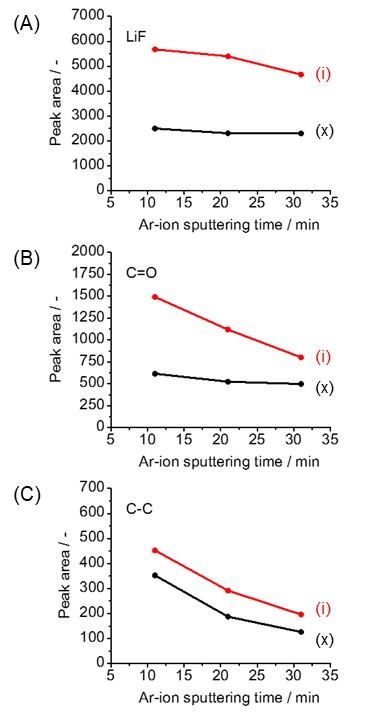
**

**Figure S10 XPS depth profiles of the elemental ratios for samples (i) and (x).** (A) LiF, (B) C=O and (C) C-C. Red and black curves indicate data for samples (i) and (viii), respectively. Ar ion sputtering for 31 min corresponded to a depth more than 238 nm.

**Table S1 Various combinations of additives assessed and the corresponding CE values.**

| **Sample** | **LiClO_4_** | **LiBOB** | **LiBr** | **DMC** | **FEC** | **CE** |
| --- | --- | --- | --- | --- | --- | --- |
| (i) | **○** | **○** | **○** | **○** | **○** | 88.6 |
| (ii) | **○** | **○** | **○** | **○** | **－** | 73.9 |
| (iii) | **○** | **○** | **○** | **－** | **○** | 78.6 |
| (iv) | **○** | **○** | **－** | **○** | **○** | 80.1 |
| (v) | **○** | **－** | **○** | **○** | **○** | 72.4 |
| (vi) | **－** | **○** | **○** | **○** | **○** | 77.6 |
|  | **○** | **○** | **○** | **－** | **－** | 71.0 |
|  | **○** | **○** | **－** | **○** | **－** | 60.2 |
|  | **○** | **○** | **－** | **－** | **○** | 72.2 |
|  | **○** | **－** | **○** | **○** | **－** | 71.0 |
|  | **○** | **－** | **○** | **－** | **○** | 77.8 |
|  | **○** | **－** | **－** | **○** | **○** | 71.2 |
| (vii) | **－** | **○** | **○** | **○** | **－** | 84.9 |
|  | **－** | **○** | **○** | **－** | **○** | 76.9 |
|  | **－** | **○** | **－** | **○** | **○** | 77.6 |
|  | **－** | **－** | **○** | **○** | **○** | 66.5 |
|  | **○** | **○** | **－** | **－** | **－** | 58.7 |
|  | **○** | **－** | **○** | **－** | **－** | 71.1 |
|  | **○** | **－** | **－** | **○** | **－** | 77.5 |
|  | **○** | **－** | **－** | **－** | **○** | 25.4 |
|  | **－** | **○** | **○** | **－** | **－** | 62.2 |
| (viii) | **－** | **○** | **－** | **○** | **－** | 82.5 |
|  | **－** | **○** | **－** | **－** | **○** | 42.9 |
|  | **－** | **－** | **○** | **○** | **－** | 72.5 |
|  | **－** | **－** | **○** | **－** | **○** | 42.8 |
|  | **－** | **－** | **－** | **○** | **○** | 79.9 |
|  | **○** | **－** | **－** | **－** | **－** | 82.3 |
|  | **－** | **○** | **－** | **－** | **－** | 78.0 |
| (ix) | **－** | **－** | **○** | **－** | **－** | 62.6 |
|  | **－** | **－** | **－** | **○** | **－** | 80.9 |
|  | **－** | **－** | **－** | **－** | **○** | 74.7 |
| (x) | **－** | **－** | **－** | **－** | **－** | 74.5 |
